# Correlation between conventional and advanced hemodynamic parameters versus serum lactate in patients with severe sepsis

**DOI:** 10.1186/cc13363

**Published:** 2014-03-17

**Authors:** M Kok, A Oei, A Karakus, H Endeman

**Affiliations:** 1 UMC Utrecht, the Netherlands; 2 AMC, Amsterdam, the Netherlands; 3 Diakonessenhuis, Utrecht, the Netherlands; 4 OLVG, Amsterdam, the Netherlands

## Introduction

The aim of this study was to determine the correlation between levels of serum lactate (SL) and conventional hemodynamic parameters (HPs) (mean arterial pressure (MAP), heart rate (HR), central venous pressure (CVP), urinary output (UP)) in patients with severe sepsis. In a subgroup, advanced HPs (central venous saturation (SvO_2_), peripheral temperature (PT), cardiac index (CI), global end-diastolic volume index (GEDI) and extravascular lung water (ELWI)) were compared with levels of SL.

## Methods

An observational prospective, single-center, pilot study was performed in intensive care (IC) of a medium-sized teaching hospital. Adult patients with severe sepsis were included and received standard goal-directed therapy (Surviving Sepsis Guidelines). Every patient received an arterial line and a central venous line in the upper diaphragm position. A subgroup received pulse contour cardiac output (PiCCO)-guided resuscitation and PT measurements. Pearson correlation coefficients (PCCs) were calculated between HPs and SL, which were measured every 4 hours for the first 48 hours after inclusion. *P *< 0.05 was considered statistically significant.

## Results

**Figure 1:**
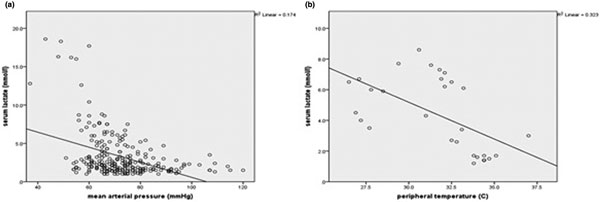
**Scatterplot of (a) MAP and SL and (b) PT and SL**.

Twenty-five patients (12 men) were included. Mean age was 68 years (30 to 93), mean APACHE II score 31 (20 to 42). The most frequent reasons for IC admission were abdominal sepsis (*n *= 11) and pneumosepsis (*n *= 7). Mean HPs (with SD and range) were respectively: MAP 73 mmHg (13, 37 to 120), HR 101 beats/minute (22, 51 to 172), CVP 12 mmHg (5, 1 to 29), UP 55 ml/hour (62, 0 to 500), SL 3.2 mmol/l (3, 1 to 18.6), CI 4.1 ml/kg/minute (1.1, 1.6 to 6.7), GEDI 871 ml/m^2 ^(210, 500 to 1,691), ELWI 11 ml/kg (5, 4 to 23), PT 32.1 C (2.8, 26.4 to 38), and SvO_2 _75% (8, 39 to 93). Relevant and significant (*P *< 0.005) PCCs between HPs and SL were respectively: MAP -0.417, HR 0.195, UP −0.237, SvO_2 _−0.204 and PT −0.569. Figure 1 shows the relation between two HPs with the highest correlation with SL.

## Conclusion

The conventional HPs MAP, HR, UP and SvO_2 _are significantly correlated to levels of SL, but clinical value might be limited due to the relatively low correlation coefficients. In a small subgroup, PT is better correlated to the level of SL.

